# Image blind detection based on LBP residue classes and color regions

**DOI:** 10.1371/journal.pone.0221627

**Published:** 2019-08-29

**Authors:** Tingge Zhu, Jiangbin Zheng, Yi Lai, Ying Liu

**Affiliations:** 1 Dept. of Computer Science and Engineering, School of Computers, Northwestern Polytechnical University, Xi’an, Shaanxi Province, China; 2 School of Telecommunication and Information Engineering, Xi’an University of Posts and Telecommunications, Xi'an, Shaanxi Province, China; 3 Key Laboratory of Electronic Information Processing Technology for Crime Scene Investigation Application, Ministry of Public Security, Xi’an, Shaanxi Province, China; Nanjing University of Information Science and Technology, CHINA

## Abstract

Forgery detection is essential to verify the integrity and authenticity of images. Existing block-based detection techniques detect forgery in the same image, most of which use similar frameworks while differ in the feature extraction schemes. These methods have high accuracy in detecting the forged regions, but the computational load is heavy when facing exhaustive search problems. This paper describes a forgery detection method based on local binary pattern residue classes and color regions. An image is divided into overlapped blocks. Local binary pattern residue classes are computed for each block. The plane formed by a dimensional and b dimensional from Lab color space is divided into 16 regions. Similar blocks are searched in the overlapped blocks with the same local binary pattern residue class and color region, then they are grouped into several suspicious regions. Finally, we analyze the multi-region relation of these suspicious regions and their areas to locate the tampered regions. The small hole is filled through the morphologic operation. The results of experiments demonstrated that our method has good performance in that it improved detection accuracy and reduced execution time under various challenging conditions. As the proposed method reduces the search range for similar blocks, it has a higher speed than exhaustive search and has comparable detection results at the same time.

## Introduction

As image processing tool is becoming more and more powerful, people can manipulate digital image quickly and easily with no obvious traces [1]. What you see on the internet is not necessarily the case. The same is true to some applications, including court certifications, newspapers, medical images, and so on. So it is becoming more and more important to verify the integrity and authenticity of digital images. Image authentication can be divided into active authentication [2] and passive authentication [3]. Active method relates to data hiding. Digital signature or watermark is embedded in an image before it is transmitted or saved, but it is seldom seen in most cases. Passive method is also known as image forensics. No prior information other than itself is required to authenticate the image. So passive methods have attracted great research interests.

**Fig 1:**
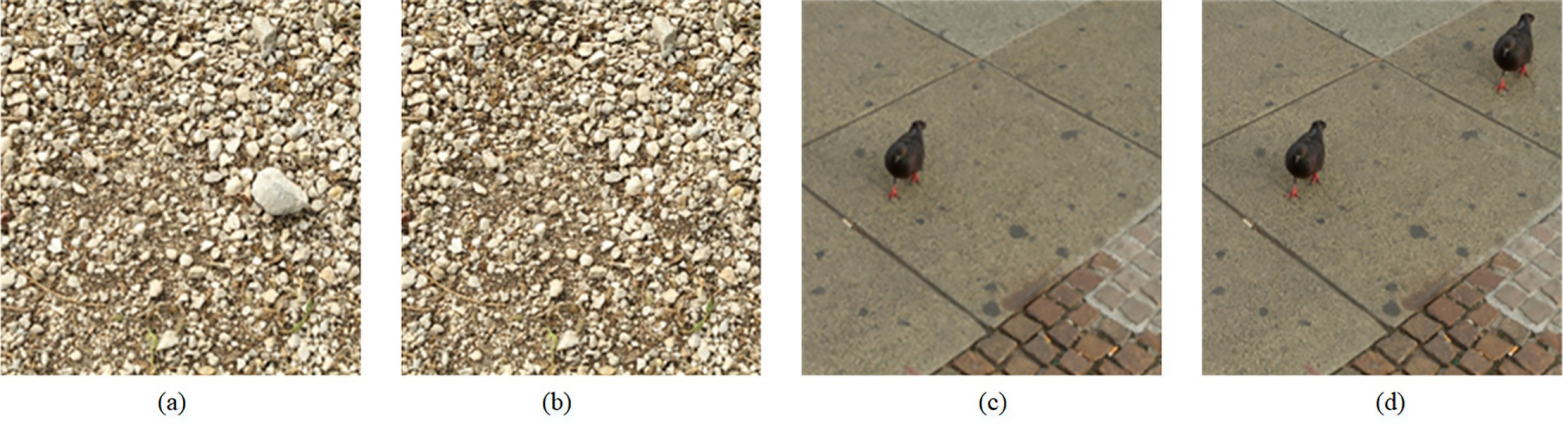
Two copy-move forgery examples. (a), (c) Original images, (b) The forgery images of (a), (d) The forgery images of (c). Fig 1 are republished from [30] under a CC BY license, with permission from Tralic D.

The easiest, yet powerful type of forgery is Copy-move. It is very common that copy-move forgery (CMF) is used to cover up the fact, for example, the object is hidden, duplicated or moved in the image. In order to be visually invisible, the constituent elements of the forged area, such as color and light, are copied from the same image. This malicious forgery is very difficult to detect with naked eyes because it is compatible with the whole image. Two examples are shown in Fig 1. A stone is removed in Fig 1(B) and a pigeon is copied in Fig 1(D), but the tampering is visually invisible. The existing copy-move forgery detection (CMFD) algorithms can be divided into two categories in the literature [3–7]: block-based detection methods [8–20] and feature point-based detection methods [20–29].

Most of the approaches are included in block-based comparison scheme, which generally detect and locate forged regions by comparing features extracted from the overlapping blocks of an image. The main difference is the strategies they used to describe the blocks, which are classified as four types [4,5], they are based on transform domain, dimension reduction, intensity and moment. In [8], forged regions are found by comparing discrete cosine transform (DCT) features of every overlapping block with the same size, and lexicographically sorting. But this method has a high feature dimension and a large amount of computation. Popescu [9] applied principal component analysis (PCA) to effectively reduce feature dimension of every block and computational cost but again does not to address geometrical transformation. In order to get more robust block representation, singular reduced-rank approximation values are calculated based on DCT in [10]. So it can resist JPEG attack with quality factors no less than 70%. In [11], Fourier-Mellin transform (FMT) was used for each block, and then the feature vector was generated by projecting FMT values to one dimension. It was able to detect cloning which is processed by up to 5% resizing attack, up to 10-degree rotation attack and JPEG compression attack with quality factors no less than 70%. In [12], a blind detection method based on Undecimated Wavelet Transforms (UWT) was proposed. It used coefficients of the UWT from every overlapping block to find similar block pairs. It can resist a few rotation attacks, scaling attack and JPEG compression attack. In [13], each block is represented by a 9-dimensional feature vector which contains information concerning the intensities of pixels. All the extracted feature vectors are then sorted by the radix sort. The method can only resist Gaussian noise and JPEG compression. Zernike moments as a feature of every block is proposed in [14,15], which is practical to transformation. The method proposed in [16] is feasible to JPEG compression using blur moments as the feature of every block. In [17], the image is divided into blocks which are circularly overlapped. Discrete Radial Harmonic Fourier Moments (DRHFMs) is used to extract the local and inner image feature of every block. For tampered images with geometrical distortions, the performance of the algorithm is very good. However, high computational time in their work is still an open problem. Because of searching similar block pairs in the whole image. In order to reduce computation complexity, the method proposed in [18] is more efficient using Fast Walsh-Hadamard transform (FWHT) to reduce the image size and Multi-Hop Jump (MHJ) to jump over some the “unnecessary testing blocks”, and it reduces the processing time greatly. However, this method is weak in resisting against the attack of transforming. In [19], the image luminance and median comparison for the blocks are used to detect tampered region. It is powerful and faster because of “Jump patch” functionality for comparison of the blocks in the Region of Suspicion. But it needs to point out suspicious regions in advance. In [20], the time complexity of the block-matching algorithm is improved by using sequential block clustering. In term of time complexity, this method is superior to lexicographical sorting.

Unlike block-based methods relying solely on block comparison, Feature point-based methods [21] are chosen to match feature points extracted from the image, such as SIFT-based methods [22–24] and SURF-based [25] methods, etc., most of these method based on these descriptors can resist some attacks, such as rotation, resizing, lighting adjustments and noise. SIFT (SURF) points are extracted, and similar points are searched in all keypoints to locate forgery regions [22–25]. In [26], SIFT feature points are matched by singular value decomposition (SVD) method which can resist geometrical transformations, so it is robust to detect high performance tampered area. But these methods can’t work in smooth regions. In order to solve this problem, Yang al et [27] can extract feature points in smooth regions to detect forgery image by using an improved SIFT algorithm. Small smooth regions can be detected in [28] because of a fused feature obtained by combining the multi-support region order-based gradient histogram for textured areas and hue histogram. The fusion method of SIFT-based and block-based is proposed in [29] to detect tampered regions, even in smooth regions. Feature point-based methods with noticeable performance are better than block-based methods in terms of robustness and computational cost. Nevertheless, feature points usually locate in textured regions, thus, plain tampered regions or small tampered regions are often failed to be detected.

In order to reduce the number of block comparisons in block-based methods and detect efficiently even under uniform image background, we proposed a novel blind method approach based on blocks for copy-move detection. We reduce time complexity by reducing the search range of matching block. Hereon, Local Binary Pattern (LBP) feature and Color Region (CR) is employed to cluster these overlapping blocks, accordingly time complexity is reduced. LBP feature [31] is used in some detection methods [32–36] before. In [32], LBP is used to represent every overlapping image block to reduce the feature dimension. In [32–36], based on LBP feature of every overlapping block, other features (SVD, DCT and gray level co-occurrence matrix) are extracted to detect the tampered regions. Exhaustive search is used in these methods mentioned above. Unlike these methods mentioned above, we first extract the texture features and color features of every block, that is, LBP feature and color components from Lab space. we define LBP residue classes (LBPRC) and Color Region (CR), matched block pairs are searched in blocks with the same LBPRC and CR. Then they are grouped into several suspicious regions and a suspicious binary map is constructed at the same time. Finally, we analyze the multi-region relation of these suspicious regions and their areas to locate the tampered regions. The rest parts of this paper are as follows. We illustrate the proposed method and its technical background in Section materials and methods. The performance of the proposed method will be evaluated by a series of experiments in Section results and discussion. Finally, in Section conclusion, summary and further research are presented.

## Materials and methods

**Fig 2:**
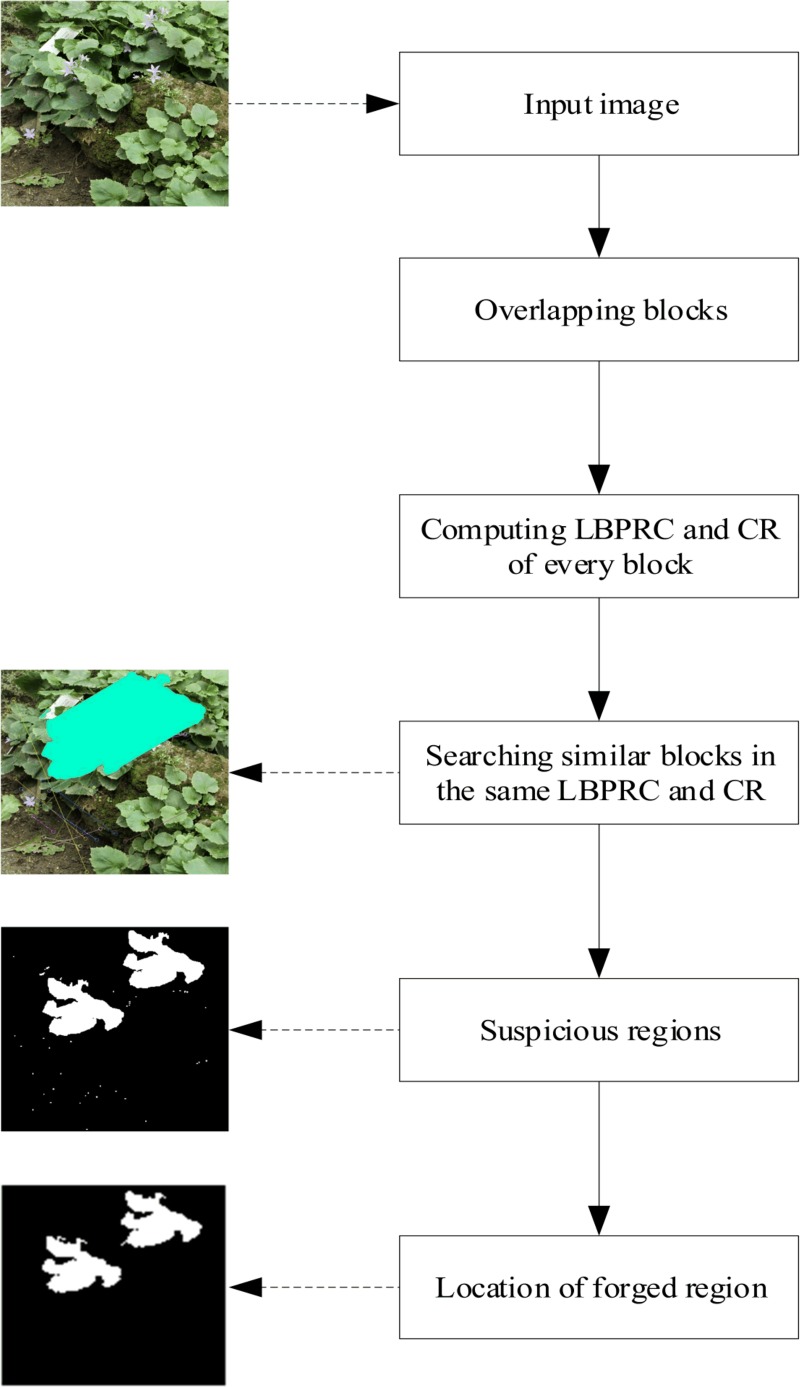
The workflow of the proposed algorithm. Fig 2 is republished from [30] under a CC BY license, with permission from Tralic D.

According to abnormal similar blocks in a tampered image, this paper proposes a novel passive forensics method. The framework of the proposed algorithm is illustrated in Fig 2, which includes two major parts suspicious region detection and tampered region location. First, we divide an image into many overlapping blocks, then LBPRC and CR of every block are computed. Instead of exhaustive search, similar blocks are searched in these blocks with same LBPRC and CR, then these matching pairs are grouped into several suspicious regions. We figure out the number of matching pairs among these suspicious regions and area of these suspicious regions. If the number of matching pairs between two suspicious regions is greater than the given threshold, and the area of the suspicious region satisfies the limited condition, which is then located as a tampered region.

For our proposed method, a *M*×*N* size image is divided into *t*×*t* size overlapping blocks. Here, *t* is an odd number. Thus there are (*M*−*t*+1)×(*N*−*t*+1) overlapping blocks. We can detect the suspicious regions based on the abnormally similar block pairs. The rest of this section will introduce several concepts for better understanding of our method.

### LBP residue class

LBP [31] is an operator of describing local image texture features, and it has remarkable invariance in the rotation and gray level. For a window of *t*×*t*, the gray values of (*t*×*t*−1) neighborhood pixels are compared with the central pixels of the window, if the value of the central pixel is less than that of the neighborhood pixel, the location of the neighborhood pixel is marked 1, otherwise 0. This produces (*t*×*t*−1) binary numbers. The vector (*a*_*n*−1_*a*_*n*−2_…*a*_1_*a*_0_) is used to stand for these numbers, that is, LBP feature of this block. The LBP values are represented by polynomials on Galois Field (*GF*(2)), 0 and 1 represent the coefficients of the LBP polynomials, then the LBP polynomial can be expressed as follows.

$$L(x)=a_{n-1}x^{n-1}+a_{n-2}x^{n-2}+\cdots +a_{1}x+a_{0},$$ (1)

where *n* = *t*×*t*−1, the power of *x* just represents the position, without the actual numerical value, so there are 2^*n*^ LBP polynomials. Let *L*_1_(*x*) and *L*_2_(*x*) be two different LBP polynomials, when they satisfy the following equations,

$$x^{k}L_{1}(x)\equiv L_{2}(x)\;mod\;(x^{n}+1),$$ (2)

where *k*≥1 and ≡’ means congruence, mod stands for modular operation. Then *L*_1_(*x*) and *L*_2_(*x*) are classified as the same LBPRC. Thus, 2^*n*^ LBP polynomials are separated into *Num* LBP residue classes according to formula 2. Hereon, *LBPRC*_*k*_(*k* = 1,2⋯*Num*) stands for all LBP residue classes.

**Fig 3:**
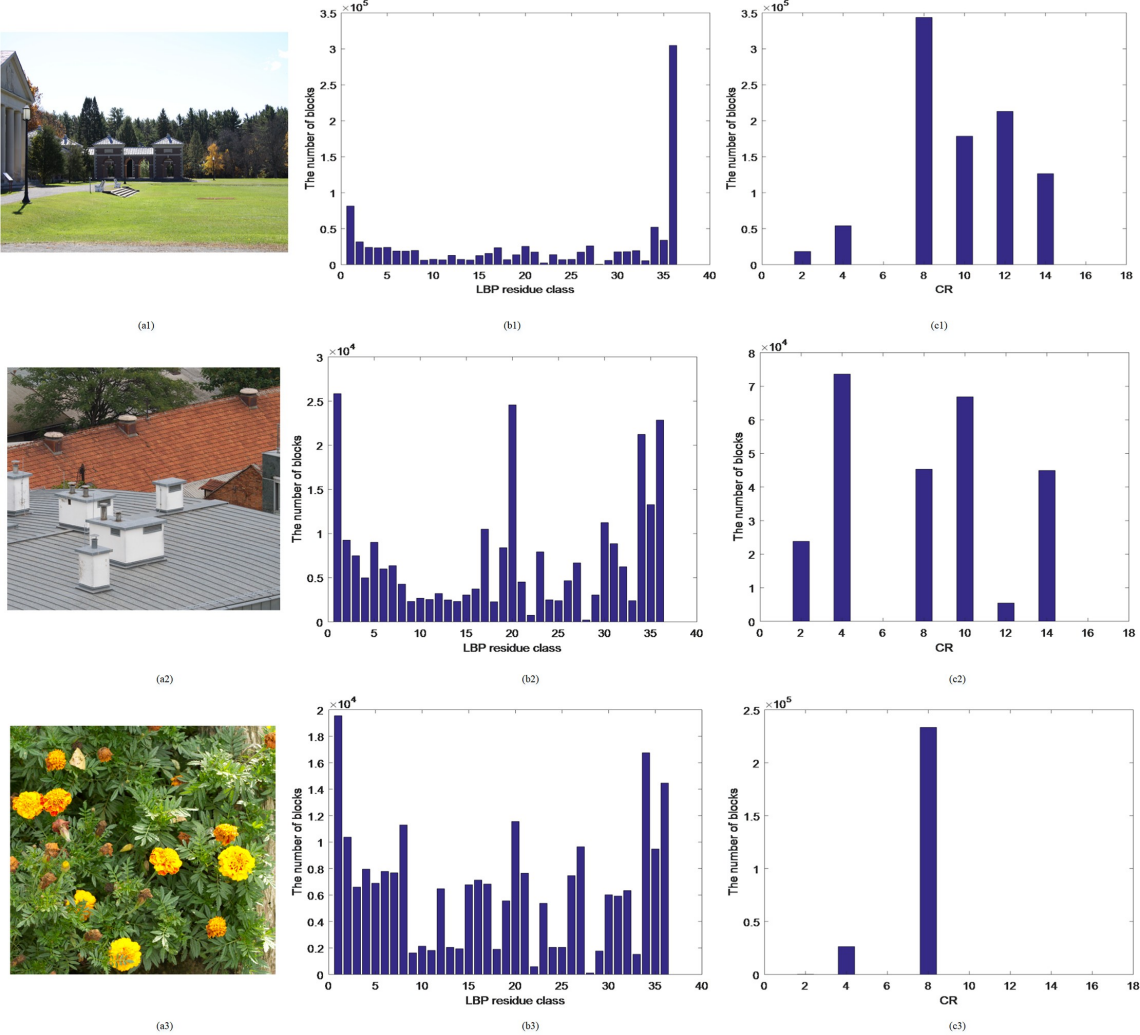
The distribution of LBPRC and CR. (a1-a3) Tampered images, (b1-b3) Distribution of LBPRC in (a1-a3), (c1-c3) Distribution of CR in (a1-a3). Fig 3(a1) from CMH dataset [37] can be downloaded from [38]. Fig 3(a2-a3) are republished from [30] under a CC BY license, with permission from Tralic D.

Each block is denoted as *Block*_*i*_,*i* = 1,2,⋯,(*M*−*t*+1)×(*N*−*t*+1). Let *L*_*i*_(*x*) denotes LBP polynomial of *Block*_*i*_. We select three images with the size of 792×1188, 512×512, and texture from simple(smooth) to complex. Let *t* be 3. And 36 LBP residue classes can be obtained by formula 2. Three examples are shown in Fig 3. The first column is tampered images. The second column is the distribution of their corresponding LBPRCs. As can be seen, when an image contains one or several smooth regions (See Fig 3(A1)), the number of image blocks belonging to one or two LBPRCs account for the great proportion of the total. Such as Fig 3(B1), the number of image blocks belonging to *LBPRC*_36_ are about 32.5% of the total. The number of image blocks belonging to other LBPRCs is much less than its number. As the area of the smooth region decreases (See Fig 3(A2)), the number of image blocks concentrated in the smooth region also decreases (See Fig 3(B2). When the texture of an image is more complex, as shown in Fig 3(A3). The biggest LBPRC contains at most 8% of the total number of image blocks. Nearly half of LBPRCs contains about 3%(1/36≈0.03) of the total number of image blocks, which is an average(See Fig 3(B3)). So the distribution of its LBPRC is relatively even.

### Color region

**Fig 4:**
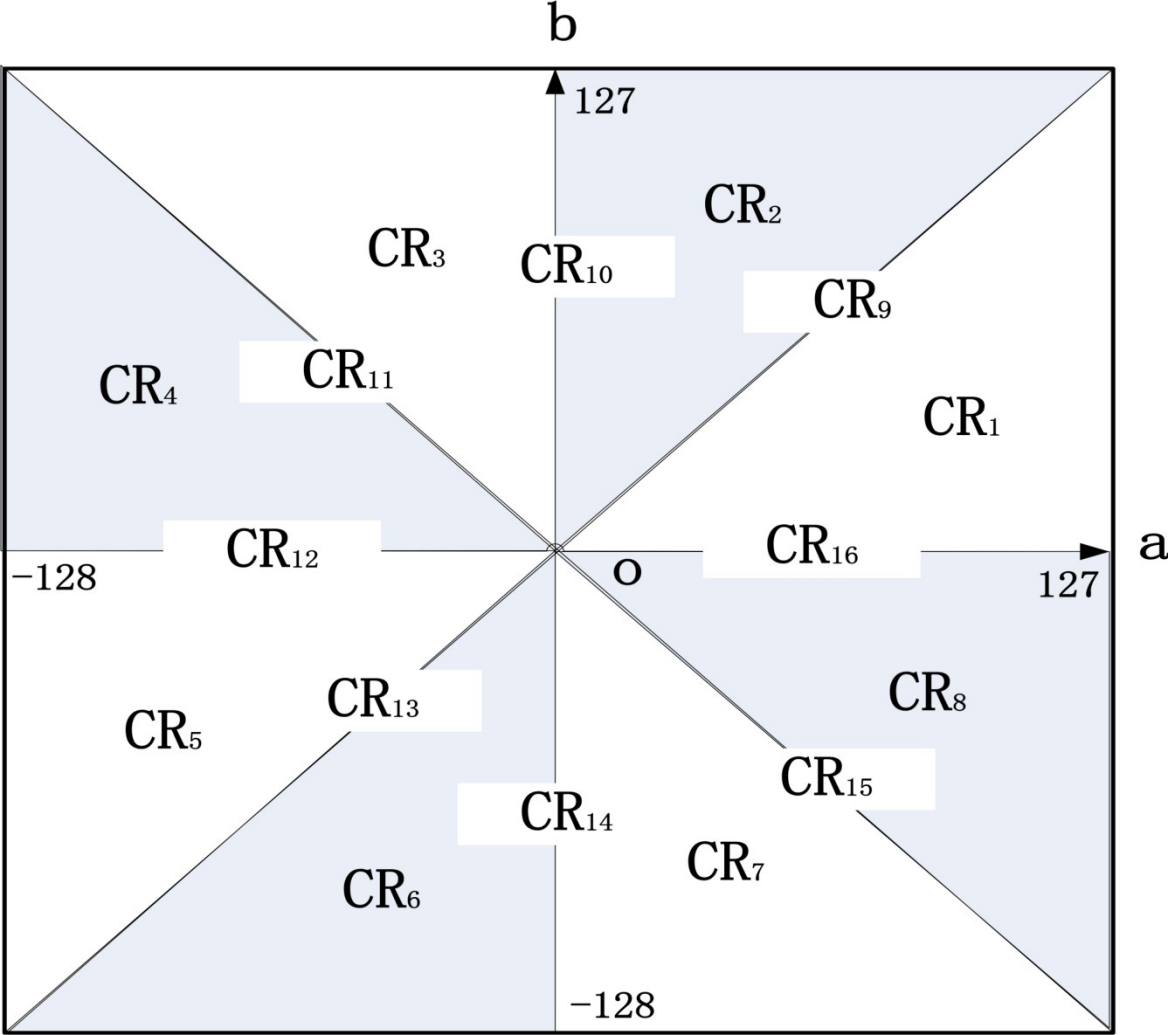
Division of *ab* plane.

All perceivable colors can be mathematically described in CIELab color space with three dimensions, *L*, *a* and *b* for lightness, green–red and blue-yellow color opponents respectively. It presents all visible colors for the human eye. So CIELab color space was a model used as a reference. Because we choose LBP feature, which Contains luminance information, so taking no account of *L* dimensional, we only choose color components, *a* dimensional and *b* dimensional, to form *ab* plane. The *ab* plane is divided into 16 regions by the following four straight lines, *b* =*a*,*b* = −*a*,*b* = 0,*a* = 0,*CR*_*j*_,*j* = 1,2…16, represents 16 color regions which are shown in Fig 4.

When a region of *ab* plane simultaneously satisfies *b*<*a*,*a*>0,*b*>0, then this region is represented by *CR*_1_. When a region of *ab* plane simultaneously satisfies *b*>*a*,*a*>0,*b*>0, then this region is represented by *CR*_2_, and so the like, we get 8 color regions. Plus four lines mentioned above, all 16 color regions are got. ${\bar{a}}_{-Block_{i}}$ and ${\bar{b}}_{-Block_{i}}$ are defined as follows.

$$\left\{ \begin{array}{l} {\bar{a}}_{-Block_{i}}=mean(a_{-Block_{i}})\\ {\bar{b}}_{-Block_{i}}=mean(b_{-Block_{i}}) \end{array}\right.,$$ (3)

where *mean*(•) is a mean value function, by which we can get the respective mean value of their components in *Block*_*i*_. $a_{-Block_{i}},b_{-Block_{i}}$ are two sets, they represent all *a* and all *b* in *Block*_*i*_, and ${\bar{a}}_{-Block_{i}},{\bar{b}}_{-Block_{i}}$ are their respective mean in *Block*_*i*_. In Fig 3, the third column shows the distribution of *CR* for three tampered images. As we can see, when an image is similar in color, the distribution of these blocks from the image is concentrated on several color regions. For example, in Fig 3(C3), there are only two color regions.

### Similarity search

Unlike exhaustive search, our method supposes that similar blocks are only searched in these blocks with the same LBPRC and CR, rather than in all overlapping blocks. If the tampered image has extensive smooth texture, most of the blocks are concentrated on several LBPRCs. As shown in Fig 3(B1), *LBPRC*_36_ contains about 32.5% of the total LBPRCs, which accounts for the highest proportion of the total. Therefore, with the limitation of the same LBPRC and CR, it significantly reduces the search range for similar block pairs. Consequently, it needs less time consumption. The similarity search is described as follows.

Step 1. An image is divided into overlapping blocks of size *t*×*t*, so there are (*M*−*t*+1)×(*N*−*t*+1) blocks.

Step 2. To calculate LBPRC and CR of every block. Let Ω_*k*_ represent all blocks in *LBPRC*_*k*_. Let Ω_*j*_ represent all blocks in *CR*_*j*_. And let Ω_*kj*_ represent the set of all blocks in the same LBPRC and CR, shown as follows.

$$\Omega_{kj}=\Omega_{k}\cap \Omega_{j},$$ (4)

where ∩ stands for the intersection.

Step 3. As recommended in [39], Exponential Fourier Moments (EFMs) are a more computable orthogonal invariant moment that has all the advantages of the circular harmonic Fourier moment, but its form is more concise. *EFM*_*i*_ is denoted as the feature vector of *Block*_*i*_, which is obtained according to the following formula.

$$EFM_{nm}=\frac{1}{2\pi b^{2}}\sum_{l=1}^{b}\sum_{t=1}^{b}Block_{i}(l,t)Q_{n}(r_{l,t})\exp(-jm\theta_{l,t}),$$ (5)

where,

$$\left\{ \begin{array}{l} Q_{n}(r_{l,t})=\sqrt{\frac{1}{r_{l,t}}}\exp(j2n\pi r_{l,t})\\ r_{l,t}=\sqrt{{\left(c_{1}t-c_{2}\right)}^{2}+{\left(c_{2}-lc_{1}\right)}^{2}}\\ \theta_{l,t}=\mathrm{ar}\;\tan\frac{c_{2}-lc_{1}}{c_{1}t-c_{2}}\\ c_{1}=\frac{\sqrt{2}}{b}\\ c_{2}=\frac{b+1}{\sqrt{2}b} \end{array}\right..$$ (6)

To take different integer for *n* and *m*, an (n, m) order moment is obtained. In this paper, *n* takes 0 and 1, so does *m*. ‖*EFM*_*nm*_‖ represents the module of *EFM*_*nm*_. So the feature vector is as follows.

$$EFM_{i}=\left\{{\left\|EFM_{00}\right\|}_{i},{\left\|EFM_{10}\right\|}_{i},{\left\|EFM_{01}\right\|}_{i},{\left\|EFM_{11}\right\|}_{i}\right\}.$$ (7)

Step 4. Block matching. *EFM*_*i*_ of *Block*_*i*_ belonging to Ω_*kj*_, as a row vector, is stored in the matrix *P*, which is lexicographically sorted. *P*' represents the sorted matrix. Adjacent blocks in *P*' are considered as suspicious block pairs. Only when their Euclidean distance is smaller than the preset threshold *d*, they are possible candidate pairs. Suppose *EFM*_*f*_ and *EFM*_*g*_ are two adjacent row vectors in *P*', when they satisfied the following formula [29].

‖pt'−pt+1'‖2<d. (8)

**Fig 5:**
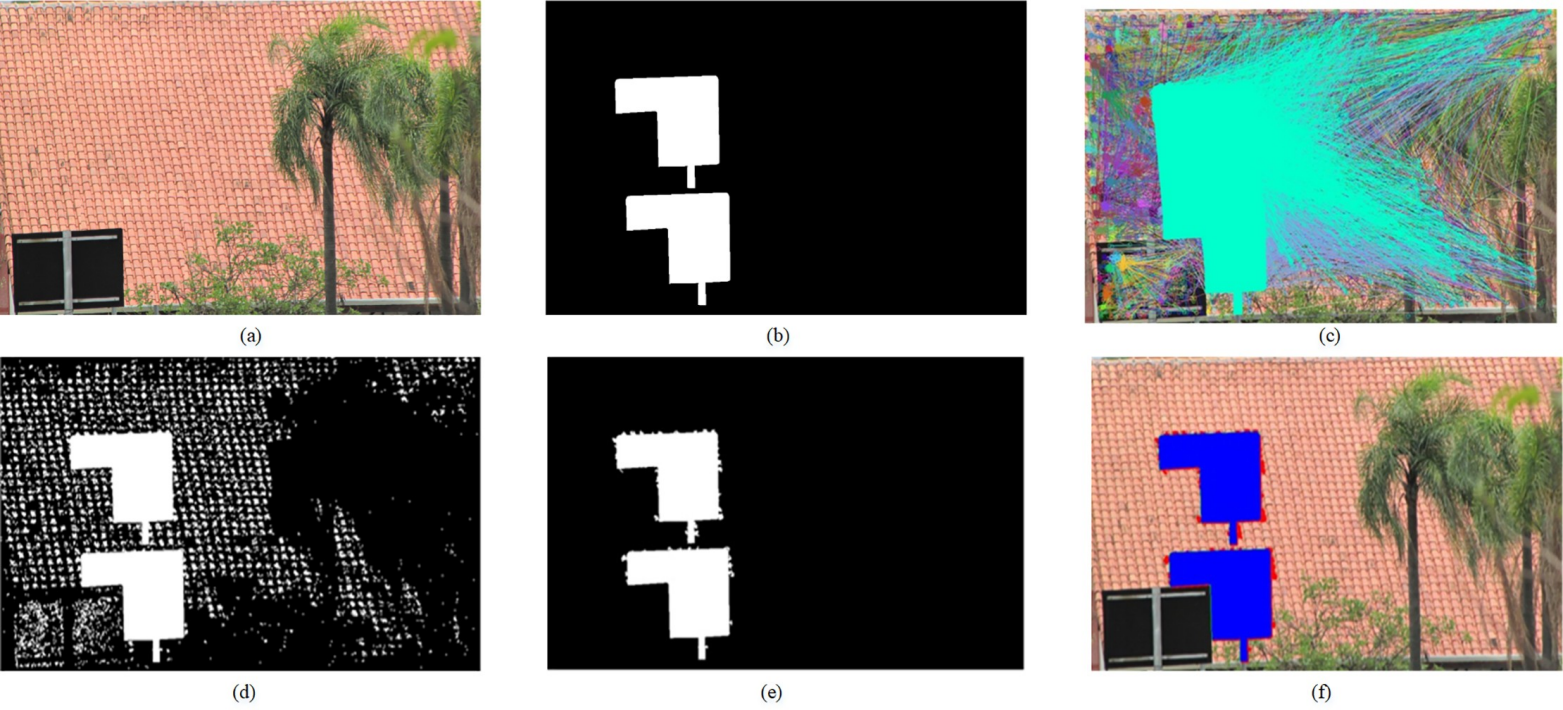
Detection example. (a) The tampered image (b) Ground truth (c) Matching block pairs (d) Suspicious regions (e) Detection result (f) Detection result shown in (a). Fig 5(A) and 5(B) from CMH dataset [37] can be downloaded from [38].

We consider the two blocks are similar. At the same time, consider the high similarity between adjacent blocks in an image, these candidate matching blocks will be removed, when the below formula holds.

$$\sqrt{{\left(x_{f}-x_{g}\right)}^{2}+{\left(y_{f}-y_{g}\right)}^{2}}<D,$$ (9)

where candidate matching blocks are indicated by coordinates (*x*_*f*_,*y*_*f*_) and (*x*_*g*_,*y*_*g*_). The threshold *D* is related to image size. In our experiment, we set *D* as 36, which is a statistical value. If the image size is larger, *D* will be larger too. Detected matching pairs are shown in Fig 5(C).

Step 5. Location and post-processing. These detected image blocks are merged into multiple regions, as shown in Fig 5(D). A suspicious region contains a certain number of image blocks. According to the process of block similarity computation, a suspicious block in a region always accompanies with its corresponding block in another region. If two suspicious regions have a similar amount of matched block pairs, we define the two regions having a strong region linkage. The region linkage can be categorized into three classes [40]: one-to-one linkage, many-to-one linkage, and self-linkage. In general, self-linkage consists of internal linkage regions, which typically occurs in uniform areas. The false positives are usually caused by self- pair linkage. Hence these regions with self-pair linkage are removed. *R* is a set, which is defined as follows.

$$R=\left\{R_{i},i=1,2\ldots N_{R}\right\},$$ (10)

where *R*_*i*_ is the *i*-*th* suspicious region, *N*_*R*_ is the total number of suspicious regions. Let *S*_*i*_ represents the number of pixels in *R*_*i*_. *S* is the set of all *S*_*i*_, which is sorted in descending order. The sorted elements in *S* are denoted as *S*_*ij*_, *j* = 1,2…*N*_*R*_. Subscript *i* and *j* indicate that *S*_*i*_ is *j*-*th* position in all *S*_*i*_. According to the characteristics of copy and paste, in general, the tampered area is more than about 0.1% of the total area. In addition, real tampered area compared with false detection is larger. Therefore, several limits are listed as follows to filter out the false positive.

$$S_{i}>\partial (M\times N),$$ (11)

$$S_{ij}>\gamma S_{i(j+1)}.$$ (12)

Here ∂ is equal to 0.01%. According to the discussion about *γ* in Section results and discussion, *γ* is equal to 0.1. When the suspicious regions satisfy these limits mentioned above, they are identified as tampered regions. In the end, the small cracks are filled by morphological operations, as shown in Fig 5(E) and 5(F).

## Results and discussion

The reliability and efficiency of our method are evaluated by the database without and with various image post-processing attacks, such as blurring, brightness changes, and contrast adjustment. Our method was implemented on a computer (Intel 2.10 GHz processor, 64GB RAM) using Matlab2016a. In the following subsection, details of the database used for evaluation, parameters setup, evaluation metrics, and robustness of the proposed approach under a variety of circumstances can be found.

### Databases

We carry out a series of experiments on two databases to test the performance of our algorithm. The first database is the CMH database provided by Silva E et al. [37]. All images can be downloaded from [38], which sizes are about 1100×800. The second database is the CoMoFoD_small_v2 database presented in [30]. This database consists of 200 image sets with 512×512. There are 40 images per transformation type, in this database, copying and pasting are used to generate tempered images. The duplicated image region(s) range from smooth to textured. The tampered regions are different in size and in quantity. In summary, the total number of images with post-processed images is 10400.

### Evaluation metric

For evaluation of our method, Recall and Precision, and F1 measurement, as defined below, are employed for pixel-level performance assessment:

$$Precision=\frac{T_{p}}{T_{p}+F_{p}}\times 100\%,$$ (13)

$$Recall=\frac{T_{p}}{T_{p}+F_{N}}\times 100\%,$$ (14)

$$F_{1}=\frac{2\times precision\times recall}{precision+recall},$$ (15)

where

*T*_*p*_ depicts the number of correctly detection pixels.

*F*_*p*_ depicts the number of wrongly detection pixels.

*F*_*N*_ depicts the number of missing detection pixels.

As a matter of fact, *T*_*p*_+*F*_*p*_ indicates the number of detected pixels and *T*_*p*_+*F*_*N*_ represents the number of forged pixels in the test image. It is obvious that the precision denotes the probability when the detected area is truly forged, and the recall is the detection probability for a forgery. *F*_1_ is a trade-off between Precision and Recall. The higher Precious, Recall and *F*_1_, the more superior performance.

### Parameters selection

In this section, the discussion of the parameters *d* and *γ* is divided into two cases: one tampered region and several tampered regions in an image. Then we discuss block size *t*.

#### ONE TAMPERED REGION IN AN IMAGE

**Fig 6:**
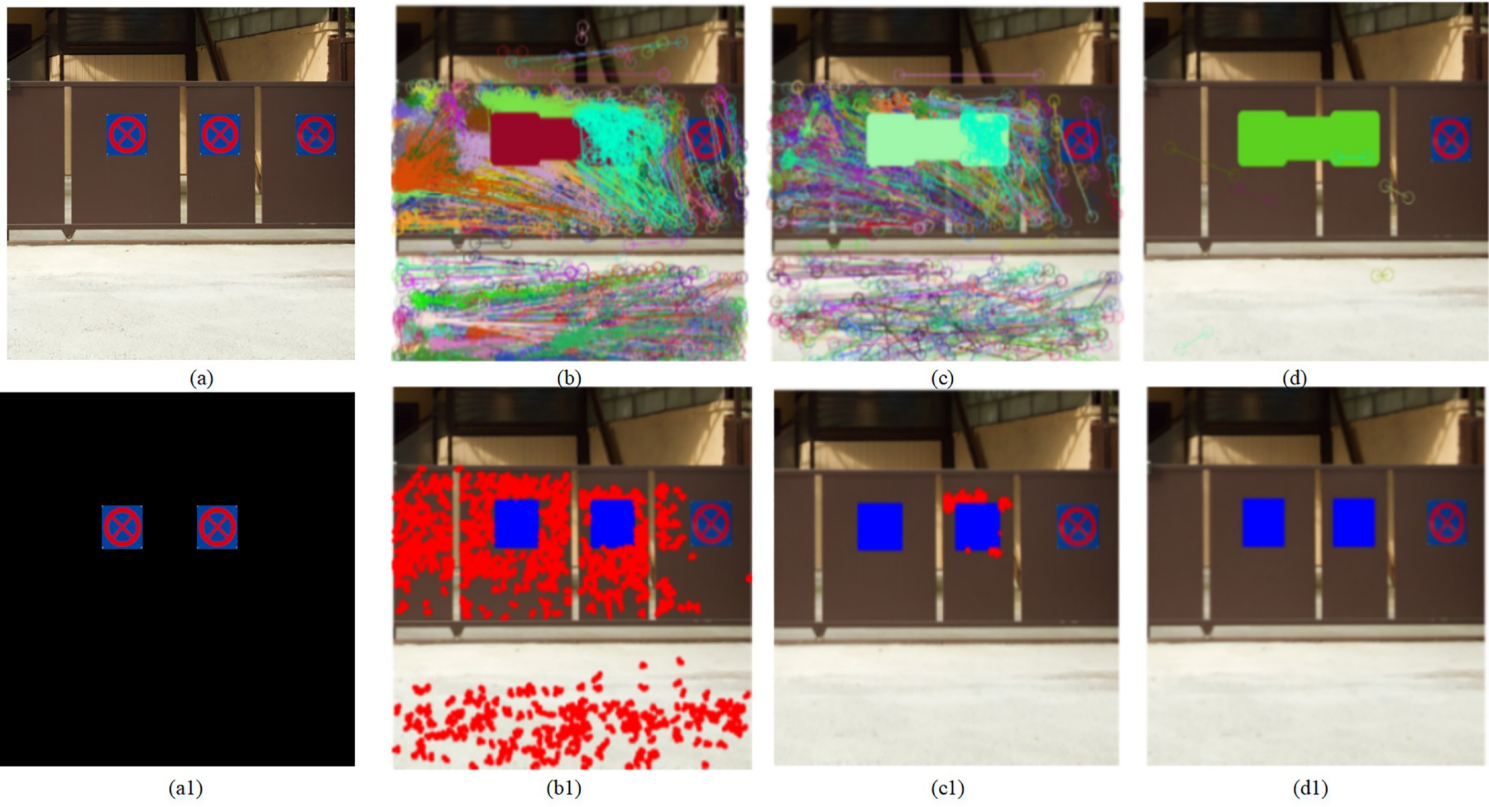
Detection results of the proposed scheme when *γ* is 0.1. (a) The tampered image, (a1) The mask image, when *d* is 0.01, 0.001 and 0.0001, (b, c and d) Detected matching pairs respectively, (b1, c1 and d1) Detection results respectively. Fig 6 are republished from [30] under a CC BY license, with permission from Tralic D.

*d* is a similarity threshold between matching blocks, which directly determines the number of matching pairs. If *d* is too large, the more matching pairs will be detected, the more post-processing time will be consumed, and even false detection will increase. While *d* is too small, the number of detected matching pairs will decrease, even the real matching block pairs will be lost. In addition, the similarity between the two blocks is high in smooth regions, which requires a smaller threshold *d*. Therefore, the choice of appropriate *d* is very crucial. We empirically set the value range of *d*, which are taken from 0.00001 to 0.1. Fig 6 shows the detection results under a different threshold *d*. Matching pairs searched in Fig 6(A) are presented in Fig 6(B), 6(C) and 6(D), where matching pairs belonging to different classes are marked in different colors. Their corresponding detection results are illustrated in Fig 6(B1), 6(C1) and 6(D1). Here, correct detection, miss detection, and false detection are marked in blue, green and red respectively. As can be seen in Fig 6, as *d* decreases, so do the number of matching pairs. But when *d* = 0.01, there are many mismatching pairs among these detected matching pair, thus false detection increase, as shown in Fig 6(B1). As the number of matching block pairs decreases, so does false detection, as shown in Fig 6(C1) and 6(D1). By a lot of experiments, we get statistically the appropriate parameter values for performance evaluation of our method, that is, *d* = 0.0001.

#### Several tampered regions in an image

**Fig 7:**
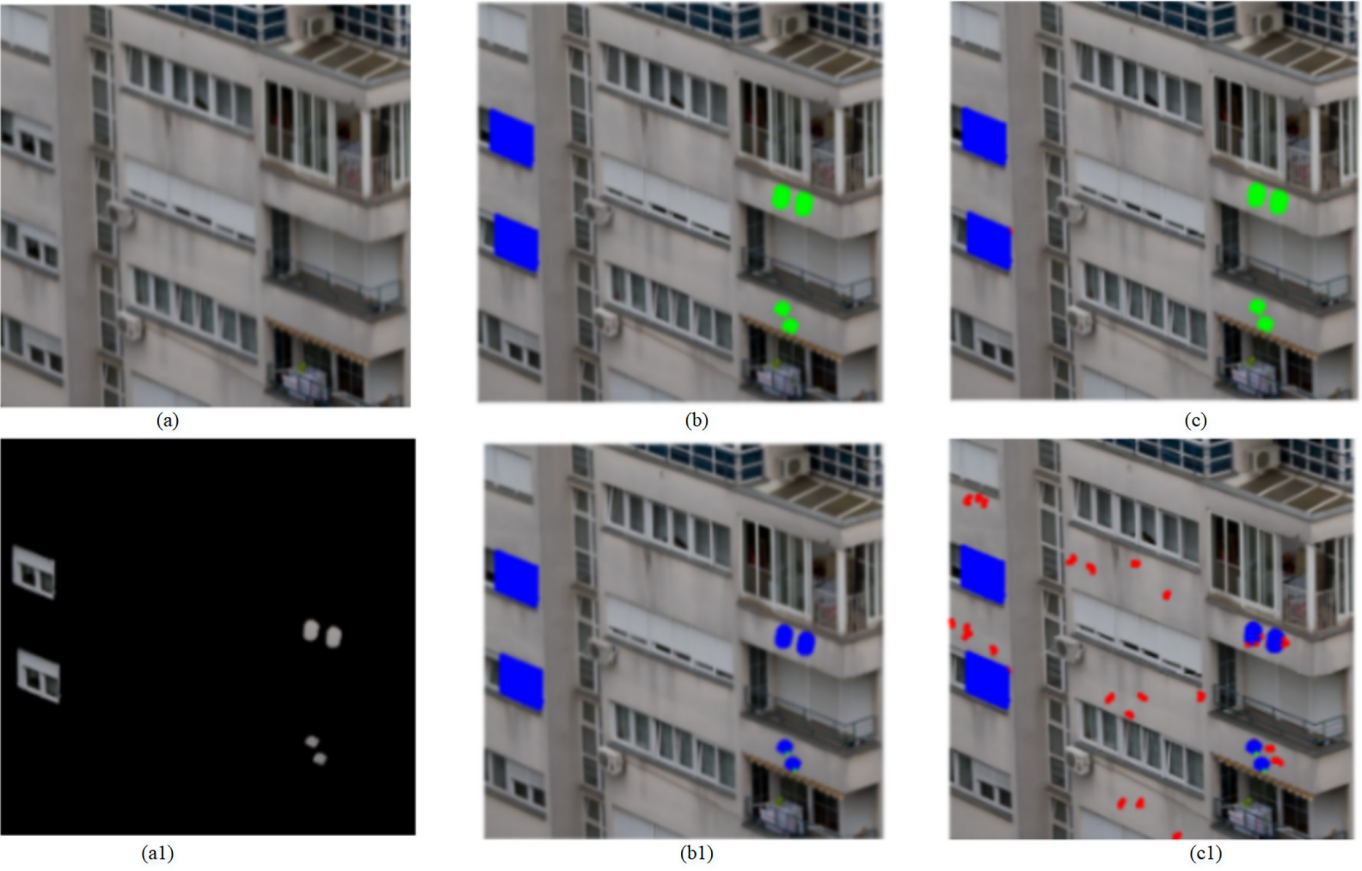
Detection results of the proposed method. (a)The tampered image, (a1) The mask image, (b) Detection result when *γ* = 0.2,*d* = 0.0001, (b1) Detection result when *γ* = 0.1,*d* = 0.0001, (c) Detection result when *γ* = 0.2,*d* = 0.001 (c1) Detection result when *γ* = 0.1,*d* = 0.001. Fig 7 are republished from [30] under a CC BY license, with permission from Tralic D.

Threshold *d* determines the number of matching pairs, and these matching pairs form tampered region(s) in an image. The larger *d*, the more matching pairs are detected, the larger region will be probably formed by matching pairs, and vice versa. When there exist several forgery regions in an image, and they differ greatly in size. If we set a larger *γ*, it will miss the small region(s) and cause false detection. Fig 7 illustrates that the different threshold *d* and threshold *γ* corresponds to their detection results. Here, red, blue, and green represent as before. As shown in Fig 7(C1), when *γ* = 0.1,*d* = 0.001, there are many false detections among these detected regions. When *γ* = 0.2,*d* = 0.001, false detections are removed, but there are many missing detections among these detected regions, as shown in Fig 7(C). By many experiments, we get statistically the appropriate parameter values for performance evaluation of our method, that is, *γ* = 0.1,*d* = 0.0001.

#### Size *t* of block

**Fig 8:**
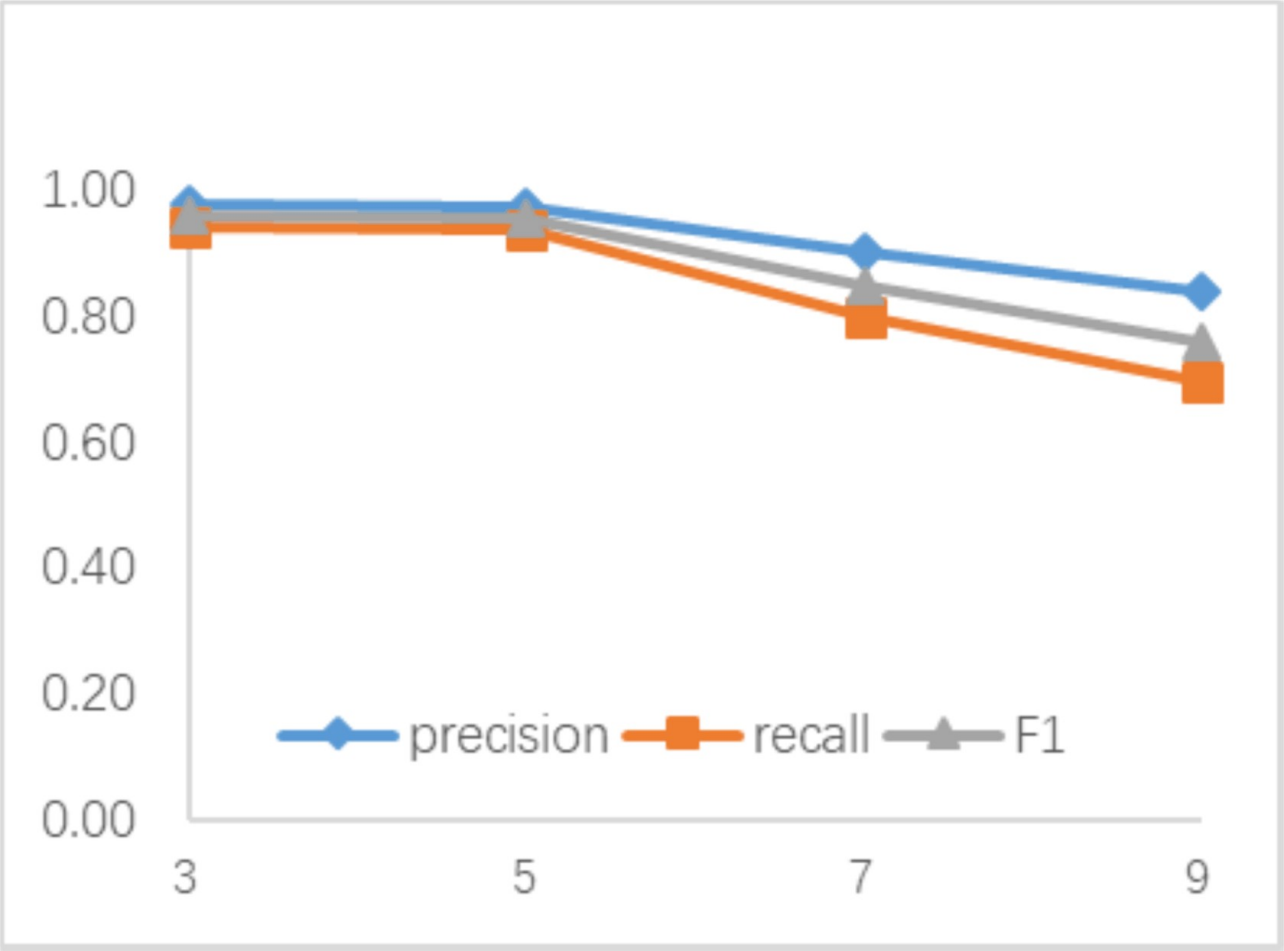
Average precision, recall and F1 curves with different block size *t*.

Reduced block size is essential to avoid missing small copy-move forged area detection, although it has increased computation overhead. In addition, the size of the image block also affects their choice for LBPRC and CR. Because similar blocks are matched in these blocks with same LBPRC and CR, detection result can be affected. Average Precision, Recall and F1 curves with different block size *t* are shown in Fig 8.

### Detection results for plain copy-move forgeries

We perform our method on two databases to evaluate the performance of plain copy-move forgery (without post-processing operation in these tampered regions). Most of the tampered image are impressively realistic, so it is difficult to distinguish true or false. The test images contain smooth or textured regions.

**Fig 9:**
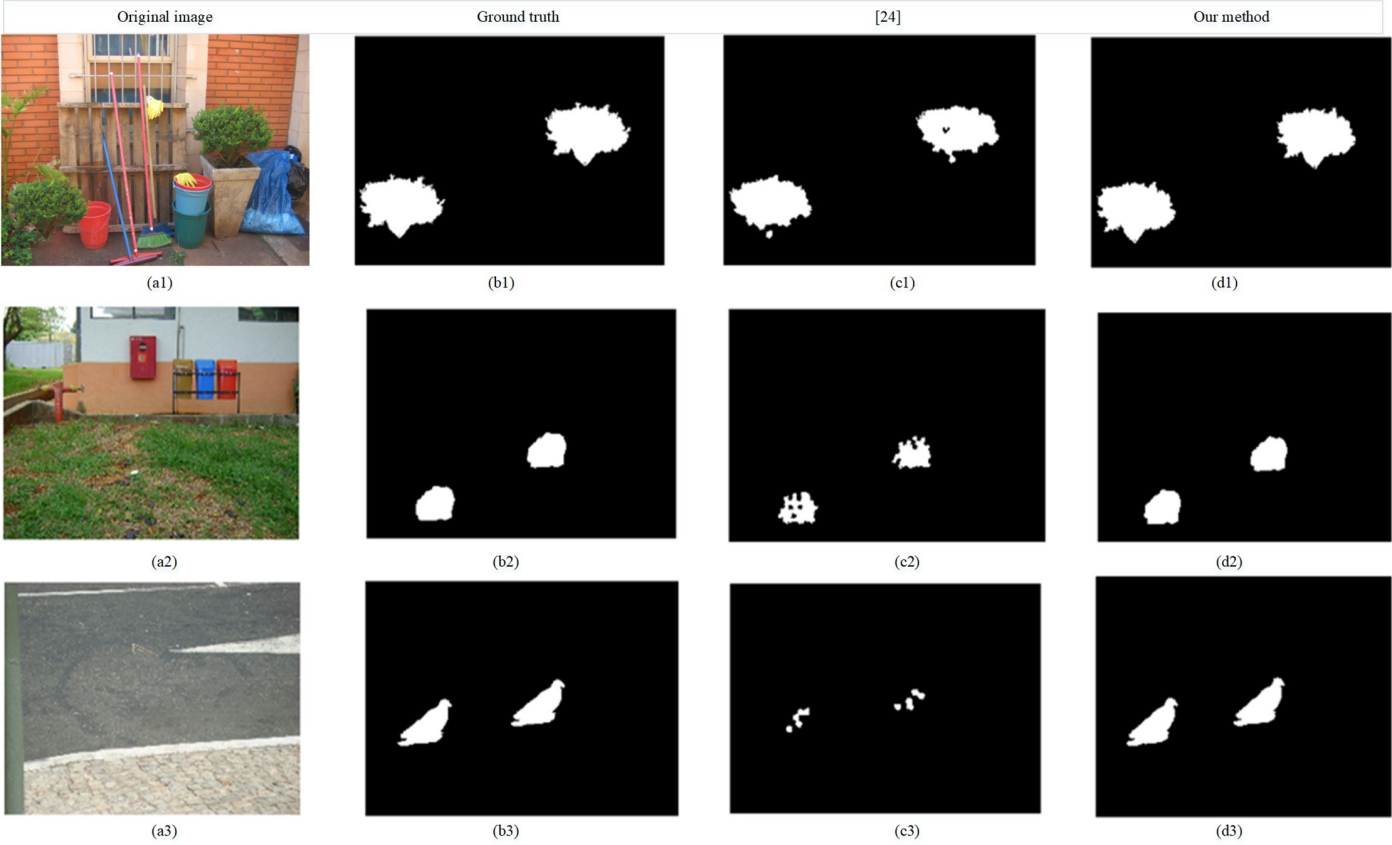
Detection results. From left to right, four columns show the test images, ground truths, and detected results using [24], our proposed scheme, respectively. Fig 9(a1-a3) and 9(b1-b3) from CMH dataset [37] can be downloaded from [38].

The first dataset contains these images with large size, most block-based methods don’t work on it because of exhaustive search and computer power. So the proposed method is just compared with method [24] on the first database. We test our method on the plain copy-move forgery. Part of detection results is shown in Fig 9. As we can see, when there are forgeries in the smooth region, the algorithm based on the method [24] can detect a portion of tampered regions, such as Fig 9(A3). It is due to that few feature points can be extracted from smooth regions. The proposed method correctly detected the copy-move forgeries in these images, as shown in Fig 9(D3). Method [24] outperforms the proposed method in terms of time-consuming, but our method has better detect results than [24] as shown in Table 1.

**Table 1:** Comparative results between [24] and the proposed method for CMH, simple cloning.

Methods	Precision(%)	Recall(%)	F_1_(%)	Run time (s)
**[24]**	86.31	87.73	87.01	121
**Our method**	89.53	93.68	91.56	989

**Fig 10:**
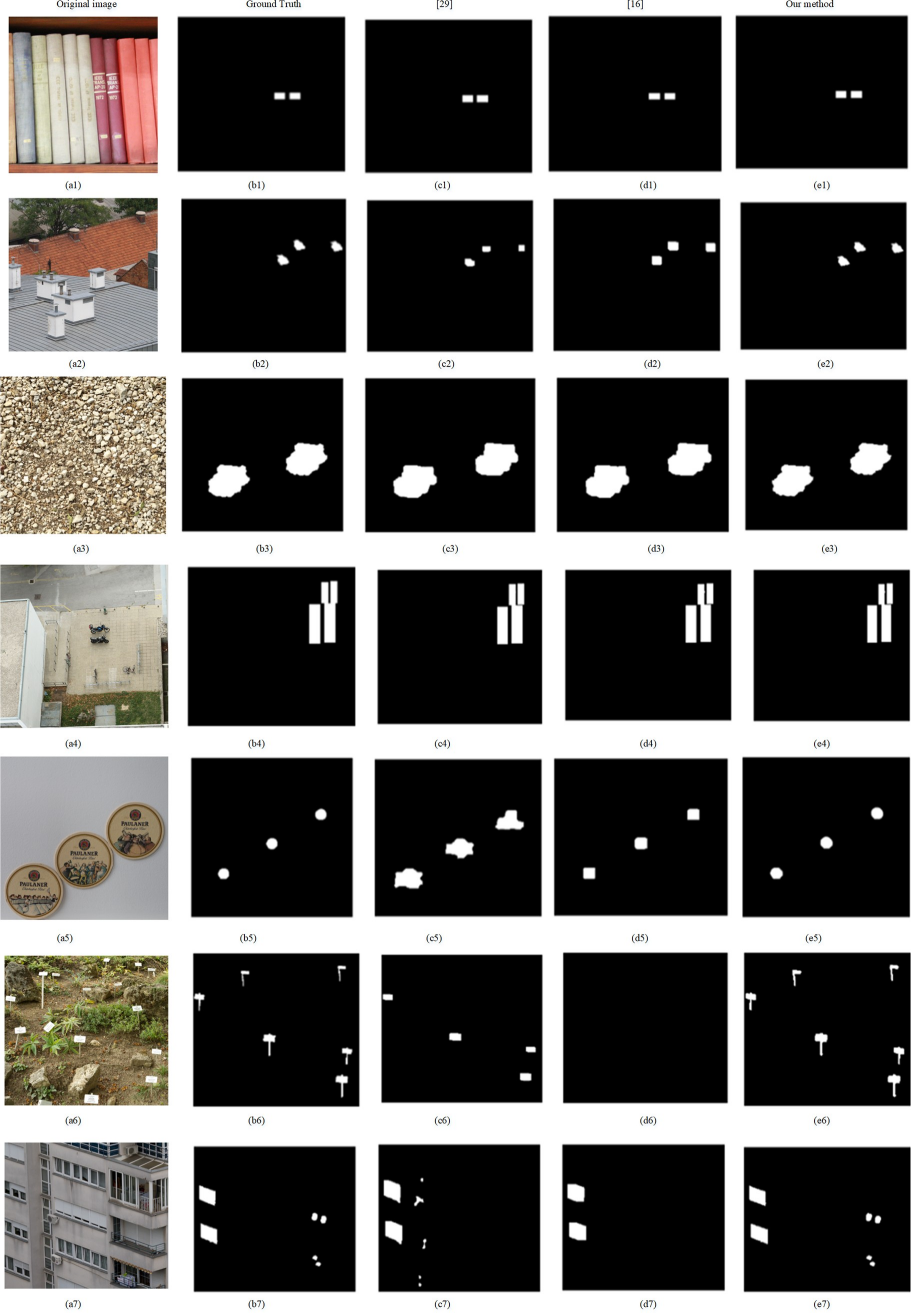
Detection results. From left to right, the five columns show the test images, the ground truth, and detected results using [29],[14],and our scheme, respectively. Fig 10(a1-a7) and 10(b1-b7) are republished from [30] under a CC BY license, with permission from Tralic D.

Image from the second database is smaller than those from the first database in size. We compare the proposed method with Zernike moment method [16] and fusion method [29] using this database. We test all images under the plain copy-move forgery. The partial detection results of the three methods mentioned above are shown in Fig 10, which are in the third, fourth and fifth columns of in Fig 10 respectively. As can be seen in Fig 10, when the tampered regions are small and smooth, Zernike-based method can only detect part of the tampered regions, as shown in Fig 10(A7), even it doesn’t work, as shown in Fig 10(A6). And fusion method misses the small smooth regions, as shown in Fig 10(A6) and 10(A7). However, the proposed approach can work effectively even in these small smooth regions. Zernike moment is more suitable for target detection, when several regions are small and smooth, where cannot be easily detected. As to the poor performance of fusion method in the small and smooth tampered region, the reason is that few feature points can be extracted, at the same time, the size of these blocks from these smooth regions is large. Because we search similar blocks in the same texture and color area, therefore, time consumption greatly reduces. Comparisons of their performance are listed in Table 2. In term of recall, precision, and F1, our method is optimal, but fusion method is better than ours in terms of time consumption.

**Table 2:** Comparative results between other methods on CoMoFoD_small_v2 database, simple clonings.

Methods	Precision(%)	Recall(%)	F_1_(%)	Running time(s)
**[29]**	86.31	84.48	87.17	153
**[14]**	87.39	84.57	88.05	3610
**Our method**	97.98	93.65	96.58	195

### Detection results for post-processing operation

**Fig 11:**
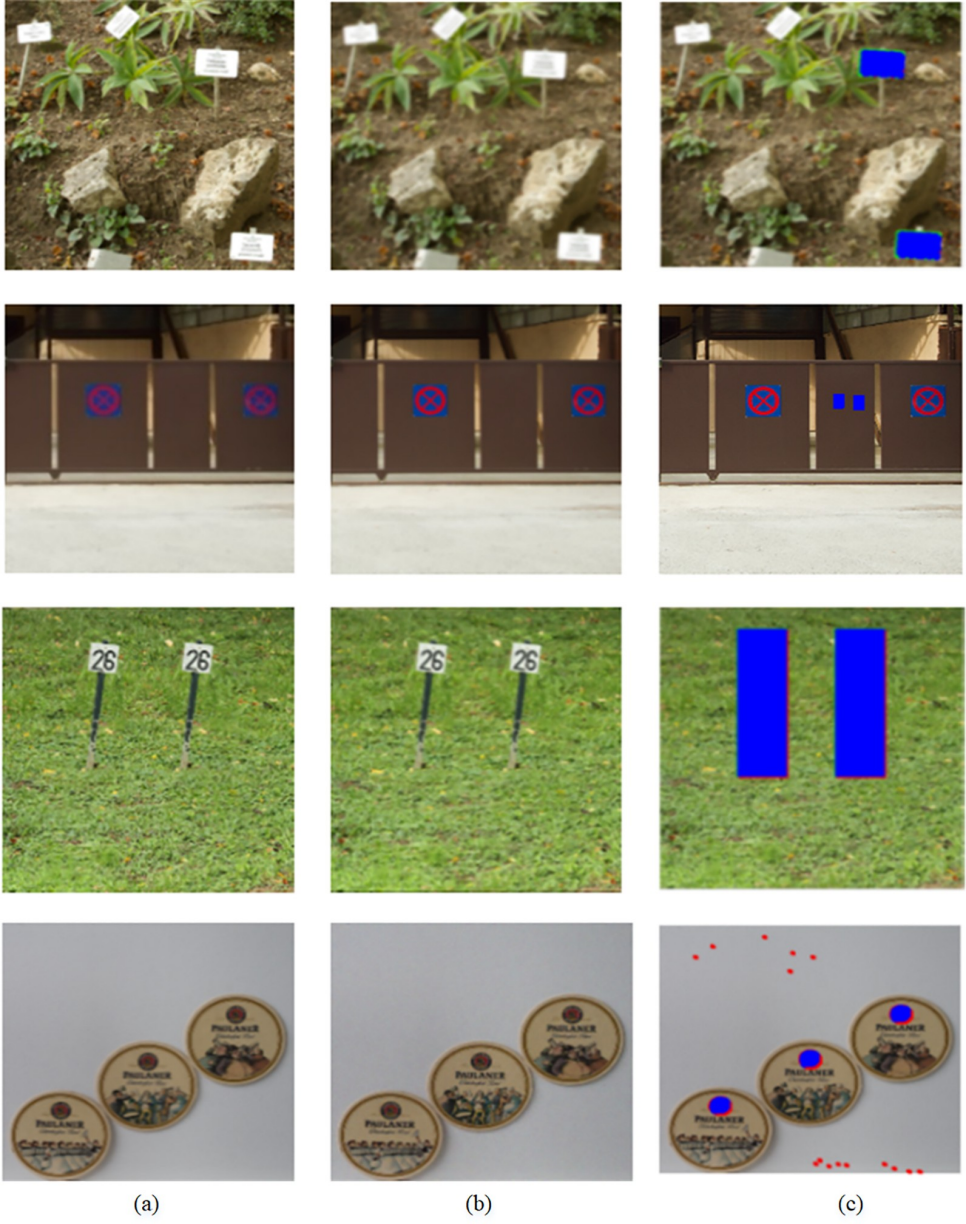
Detected results under conditions of blurring, contrast adjustment and brightness change. (a) Tampered images, (b) Tampered images with blurring, contrast adjustment, brightness changes and noise from top to bottom (c) Detection results shown in post-processed images, Here, red, blue and green represent as before. Fig 11 are republished from [30] under a CC BY license, with permission from Tralic D.

The CoMoFoD dataset provides post-processed images on which each operation is done at three different parameters. A series of experiments evaluate the performance of the proposed method against various post-processing operations which includes blurring, contrast adjustment, and brightness changes. Table 3 presents the details of the parameters used for three post-processing operations. Fig 11 shows the results using the proposed method, under conditions of image blurring, contrast adjustments, brightness changes, and noise. When an image is operated by blurring, contrast adjustments, and brightness changes respectively at different levels. Here, we take an average of three levels for each post-processing operation. Detection results are listed in Table 4 and Table 5. The proposed method is compared with [16], [24], [27] and [29]. It is obvious that the proposed method has better performance in term of brightness changes and contrast adjustments. Because images have applied conversion from RGB space to Lab space. We only extract their color feature for color segmentation, to a certain extent it reduces the effect of image brightness. The proposed approach is comparable to other methods in terms of F1 when the tampered image is blurred. But the proposed method is the worst when the noise attack is large.

**Table 3:** Parameters used for post-processing in CoMoFoD dataset.

Parameters	Brightness Changes Lower and upper bounds	Contrast Adjustments Lower and upper bounds	Blurring Variance	Noise averaging filter
**Level 1**	0.01,0.95	0.01,0.95	0.009	3×3
**Level 2**	0.01,0.9	0.01,0.9	0.005	5×5
**Level 3**	0.01,0.8	0.01,0.8	0.0005	7×7

**Table 4:** Comparative results for post-processing operations.

Methods	Brightness change			Contrast adjustment		
	Precision (%)	Recall (%)	F_1_ (%)	Precision (%)	Recall (%)	F_1_ (%)
**[16]**	75.24	70.27	72.52	70.24	73.21	70.12
**[24]**	76.25	72.92	73.91	75.49	73.21	76.09
**[27]**	89.12	83.1	86	86.8	85.2	85.99
**[29]**	74.31	69.89	72.09	70.19	73.28	71.05
**Our method**	93.65	85.71	87.78	96.58	90.31	88.10

**Table 5:** Comparative results for post-processing operations.

Methods	Blurring			Noise		
	Precision (%)	Recall (%)	F_1_ (%)	Precision (%)	Recall (%)	F_1_(%)
**[16]**	68.41	70.02	68.17	80.33	77.49	78.18
**[24]**	73.02	64.53	68.21	77.49	72.21	76.09
**[27]**	90.1	88.7	89.39	83.5	89.3	86.3
**[29]**	70.14	65.61	66.79	81.34	79.73	80.26
**Our method**	71.65	68.28	69.01	62.05	50.81	55.48

### Search space for blocks matching

Algorithm complexity depends largely on search space for feature point (block) matching. The larger the search space, the higher the hardware requirements, the more time-consuming. For point-based methods, because an image has different texture features, feature points extracted in an image range from a few hundred to several thousand, such as SIFT, SURF, etc. For block-based methods, in general, an image is divided into (*M*−*t*+1)×(*N*−*t*+1) overlapping blocks, such as classic Zernike moment, DCT, etc. Then similar points (blocks) are searched in all feature points (blocks).

The total number of overlapping blocks is much larger than the number of feature points, so the search space for point-based methods is much smaller than that for block-based methods. Accordingly, the point-based methods require low hardware and time-consuming. In this paper, we set *t* = 3, according to color and texture, all blocks are divided into at least 1 category and at most up to 36×16 categories. Similar blocks are searched in these blocks belonging to the same category. Compared with general block-based methods, the proposed method greatly reduces the search scope, the complexity of the algorithm, hardware requirements and time-consuming.

In our experiments, for the first database, most block-based methods don’t work, such as Zernike moment, DCT and so on. The proposed method and point-based method can do. Compared with the SIFT-based method, though the proposed method takes more time (as shown in Table 2), it has higher accuracy. In addition, when images are large and their textures are smooth, the proposed method doesn’t work, it can detect a majority of images from the first database. In the second database, the proposed method is superior to the method based on Zernike moment in speed due to its smaller search space. Especially when image texture and color are abundant. Here, Fusion method based on block and point employs block matching in smooth regions, so Our method is are comparable with it in term of speed when the image texture and color are abundant (as shown in Table 2). When there are most smooth regions in an image, the fusion method is a little slower than the proposed method because its search space for block matching is larger than our method’s.

## Conclusion

**Fig 12:**
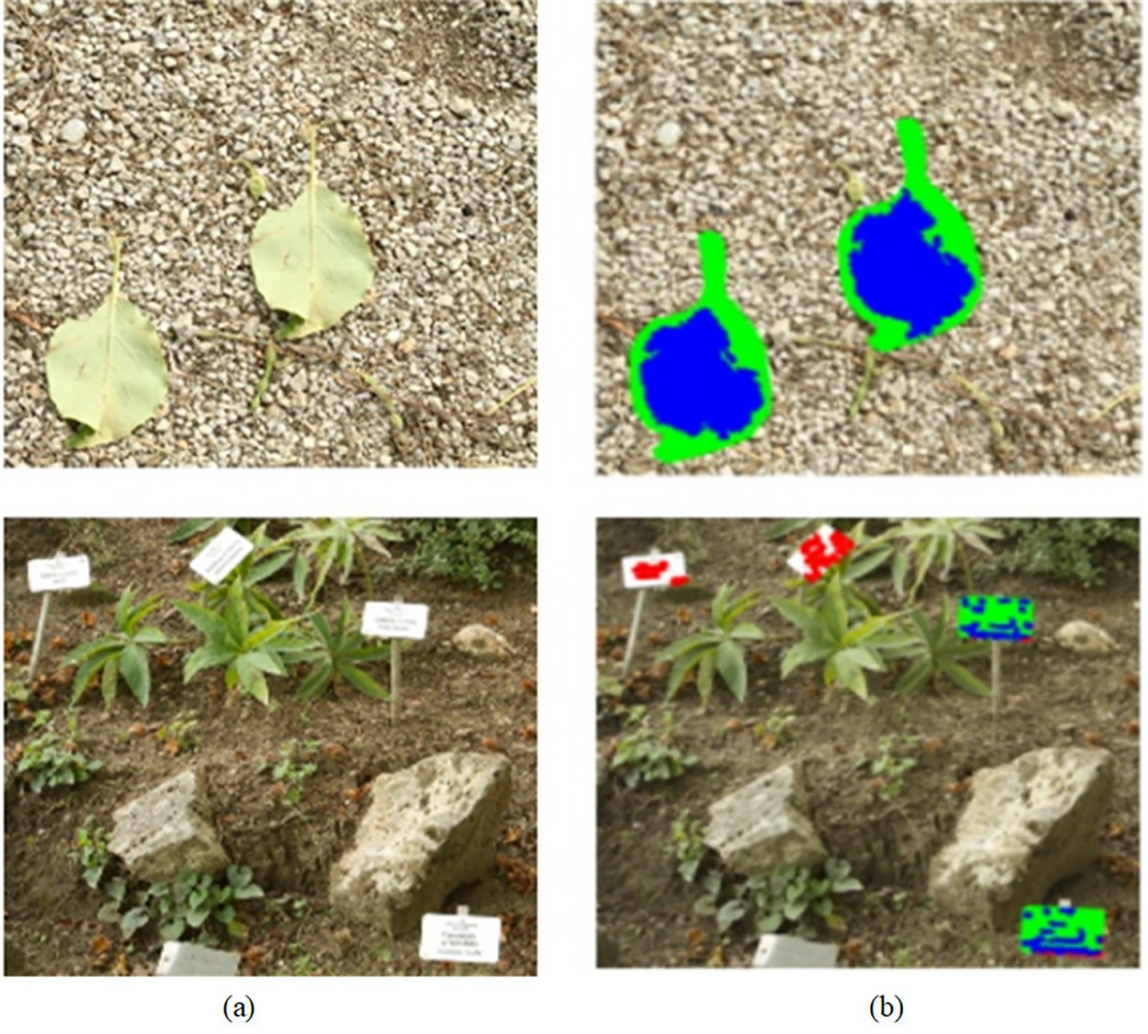
Detection results for joint attack. (a) Forged images, (b) Detection results when the tampered region is rotated and the tampered images are attacked by noise or blurred. Fig 12 are republished from [30] under a CC BY license, with permission from Tralic D.

This paper presents a novel image forgery detection approach based on LBPRC and color regions. An image is divided into several regions with the same texture and color, in which similar blocks are searched. So search space for similar blocks are decreased and time-consuming is greatly reduced, at the same time, tampered regions are effectively located. Because the proposed algorithm is less resistant to scaling and rotation attacks. So we will investigate how to speed up block matching and the detection accuracy of rotation and scaling in our future work. In addition, most blind detection algorithms can only detect one kind of tampering operation or several tampering operations, and can’t detect many tampering operations. So do the proposed method, as shown in Fig 12. In the last two years, blind detections based on deep learning [41] were proposed, which used deep learning to analyze the change of image features. We consider using deep learning [42,43] to analyze the changes in image features after various tampering in order to achieve the purpose of detecting various image tampering methods. As mentioned this will also be the direction of our future research.
